# Knowledge, attitudes, practices, and the effects of COVID-19 among the youth in Kenya

**DOI:** 10.1186/s12889-021-11067-2

**Published:** 2021-05-30

**Authors:** Evalin Karijo, Sylvia Wamugi, Samuel Lemanyishoe, Jenny Njuki, Faith Boit, Vania Kibui, Sarah Karanja, Timothy Abuya

**Affiliations:** 1 Y-ACT, Youth in Action, Amref Health Africa, Nairobi, Kenya; 2 Population Council, Nairobi, Kenya

**Keywords:** COVID-19, Youth, Knowledge, Attitudes, Practices, Sexual and reproductive health, Socio-economic

## Abstract

**Background:**

Cases of the Corona Virus Disease of 2019 (COVID-19) in Kenya have continued to increase rapidly, since the first case in the country was confirmed in March 2020. In the wake of the pandemic, the health and socio-economic challenges experienced by the youth in Kenya are likely to be elevated. We assessed knowledge, practices, perceived risk of infection, adoption of recommended behaviour and the effects of COVID-19 among the youth in Kenya.

**Methods:**

A cross sectional descriptive study was conducted between April 30th to May 7th, 2020 through a combined online survey and phone interviews. A total of 2156 youth across all 47 counties in Kenya completed the responses to the study questions. All survey responses analyzed using Stata version 15 were tabulated by gender, age, and education level to generate basic descriptive tables and tested for differences by category using chi-square tests. Where applicable, linear and logistic regression analysis model was conducted using covariates such as employment status, gender, and education level.

**Results:**

Knowledge on symptoms of COVID-19 was generally high. Female respondents were more likely to identify more symptoms correctly compared to men (*p* < 0.001). However, youth reported very low levels of anyone being at risk of infection (7.1%). Most youth have adopted behavior necessary to slow down the infection. There were generally very low reported levels of inability to access health services related to sexual and reproductive health. About 50.0% of respondents reported significant decline in income during the pandemic period, nearly a third reported living in fear while 26.5% reported feeling stressed.

**Conclusion:**

There was high knowledge of COVID-19 symptoms, preventive strategies, and high adoption of preventive practices. Strategies to sustain behaviors positively adopted among young people will be critical to reduce the spread of COVID-19. Despite the low reported rates of inability to access sexual and reproductive health, response measures should include strategies that facilitate continuity of services among young people. The reported social effects of the pandemic show the need for interventions to meet the health and socio-economic needs of the youth and minimize the long-term consequences of the pandemic.

**Supplementary Information:**

The online version contains supplementary material available at 10.1186/s12889-021-11067-2.

## Background

Coronaviruses are a family of respiratory viruses that cause common cold, Middle East Respiratory Syndrome (MERS) and Severe Acute Respiratory Syndrome (SARS) [1–5]. The coronavirus disease of 2019 (COVID-19) was isolated and referenced as severe acute respiratory syndrome coronavirus 2 (SARS-CoV-2) [6]. The disease has since become a global pandemic [6–8] forcing countries to implement measures to slow the spread that has impacted global economies [9]. Initial modelling studies indicated that the risk of importation to African countries was heterogeneous with countries like Egypt, Algeria, and South Africa having the highest importation risk and moderate to high capacity to respond. Nigeria, Ethiopia, Sudan, Angola, Tanzania, Ghana, and Kenya had moderate risk with variable capacity to respond [10]. Since then, the virus has spread to many African countries with 54 of 55 African Union Member States reporting over 100,000 cases and 3100 deaths by May 2020 [11].

In Kenya, since the announcement of its first case of COVID-19 in early March [12], there was a rise in COVID-19 cases, estimated at 320 at the start of this study rising to 607 within a week [13]. In response, the Kenya government implemented a mix of public health response measures, including messaging to create awareness on preventive measures, such as the use of masks, practicing hand hygiene and social distancing. Additionally, there were international travel bans and cessation of movements in and out of areas that exhibited high rates of infections. The government also implemented a dusk to dawn curfew in all 47 counties and shut down public places including schools, churches, and other social gatherings, to slow down the spread of the virus.

Despite these measures and given the risk of poor outcomes among older persons and those with underlying conditions [6], young people are likely to contribute to the spread of the virus, given their high population and mobility. For example, 75 % of the 47.6 million Kenyans (35.7 million) is under the age of 35 years, while young people aged 18–34 years constitute 29% (13.7 million) of the total population [14]. Although young people present an opportunity to drive the country’s economy, they face numerous health and socio-economic challenges [15]. For example, adolescents in Kenya experience poor sexual and reproductive health (SRH) outcomes [16]. The 2014 Kenya Demographic and Health Survey shows that one in every five teenage girls between the ages of 15–19 has begun childbearing, while the contraceptive prevalence rate among sexually active unmarried girls aged 15–19 years and 20–24 years is 49 and 64% respectively [17]. The country’s estimates for human immunodeficiency virus (HIV) showed that young women aged 15–24 years accounted for a third of all new HIV adult infections [18]. Other than poor health outcomes, the youth unemployment rate is estimated at 35%, compared to the overall national unemployment rate of 10%. Furthermore, 80% of unemployed Kenyans are below 35 years old [19].

Based on experiences of previous outbreaks in Africa, such as Ebola, failure to contain the virus is likely to overwhelm health systems and increase mortality from malaria, HIV and acquired immunodeficiency syndrome (AIDS) and tuberculosis [20, 21]. The pandemic is also likely to strain access to youth friendly services and SRH information. Shortages of medications such as contraceptives, antiretroviral drugs for HIV/AIDS and antibiotics to treat sexually transmitted infections (STIs), due to disruptions in supply chain is likely to affect women and girls [22]. As governments provide solutions to fight the pandemic, its impact on a country’s health systems and the economy at large needs to be considered from the outset to avoid disruption. To guide immediate pandemic response, we conducted a study to establish knowledge, practices, perceived risk of infection, adoption of recommended behavior and the effects of COVID-19 among the youth in Kenya.

## Methods

This was a cross sectional descriptive study conducted from April 30th to May 7th, 2020 using a combined online survey and phone interviews. Out of 3414 youth who were targeted for the study, a total of 2156 youth aged 18–35 years across all 47 counties in Kenya completed the survey or phone interviews fully. Respondents were drawn from Amref Health Africa’s Y-ACT, Youth in Action platform, other stakeholders’ youth platforms, youth-referrals via short message service (SMS) and social media. The study was conducted under Y-ACT, Youth in Action’s national network of youth, an initiative of Amref Health Africa established in 2017. Y-ACT represents the voice of young people aged 18–35 years, advocating for policy changes in SRH and Gender Equality. Over the last 3 years, Y-ACT has created and mobilized an online advocacy movement of over 3414 youth advocates across all 47 counties in Kenya. Y-ACT has catalyzed key policies in Kenya including the Adolescent SRH Policy in Nairobi County, gender protection policy in Kilifi County, adoption of meaningful youth engagement with national policy makers and prioritization of youth SRH issues in several counties. Since the survey was not assessing any impact, we targeted all the 3414 and any other eligible youth who consented to participate in the survey.

### Data collection activities

A set of 40 questions were adopted from previous COVID-19 related studies in Kenya [23]. Six Kenyan enumerators were trained remotely on the survey instrument using Zoom platform. The survey instrument was piloted with young people who were not part of the Y-ACT platform. The questionnaire was then administered among the youth networks. Before administering the questionnaires, the content of the study and the eligibility criteria were explained through various platforms, including SMS, the survey email, and online discussions. Thereafter a link with the tool, which included an online written consent section, was circulated to all participants in various platforms including Y-ACT’s and other social media platforms. Once the online link was circulated, the team of six trained research assistants (RA) followed up with phone-based interviews to urge the youth to complete the survey. The RAs also interviewed those who were not able to complete the online tool on their own – a total of 118 respondents were interviewed on phone. The RAs were selected from a team that had extensive experience working with vulnerable populations. A written online consent to participate in the study was included in the survey tool, for participants to give consent prior to responding to the questions. The informed consent for the phone interviews was provided verbally by the respondents and transferred to the written consent section of the survey tool on their behalf alongside their survey responses. The same survey questionnaire was used for the phone interviews. During data collection, participants had an option of terminating the study at any time. No financial or other incentives were given for taking part in the study.

The survey aimed to collect information on the level of knowledge on signs and symptoms of COVID-19, commonly used channels of information on COVID-19, adoption of preventive practices, and the effects of COVID-19 among the youth, focusing on access to specific health care services and other social and economic effects of the pandemic, as informed by literature review of other epidemics/pandemics and effects on SRH, health service delivery and socio-economic factors. The tool also covered the youth’ perceived risk of infection, fears or concerns regarding the outbreak. (See supplementary file 1). We received expedited ethical approval for the rapid survey, to provide timely evidence that was needed to guide community engagement activities. The survey was approved by Amref Health Africa Ethics and Scientific Review Committee (ESRC) under the reference: Amref-ESRC P798/2020.

### Data processing and analysis

The collected data was exported to Stata version 15 for analysis. The data was cleaned in preparation for descriptive analysis. All survey responses were tabulated by gender, age and education level to generate basic descriptive tables, and tested for differences by category using chi-square tests or t-test where applicable with a significance level of 0.05. Where applicable, multivariate linear and logistic regression analysis was conducted comparing various outcome indicators with covariates such as employment status, gender, and education level. All datasets analyzed during this study are included in the supplementary files (see supplementary file 2).

## Results

### Characteristics of study participants

A total of 2177 youth were reached through the online survey. Twenty-one (21) did not complete all the questions, hence data for a total of 2156 youth was used for the analysis. Table 1 shows the characteristics of the study respondents. The average age of participating youth was 26 years. Most of the respondents were aged between 25 and 29 years and 74.2% had completed higher education. In terms of marital status, 69.9% were single and nearly half of the respondents (49.7%) were not employed.

**Table 1:** Characteristics of the study respondents

Characteristics	Female		Male		Total		***P*** values
	***n*** = 1083	(%)	***n*** = 1073	(%)	***n*** = 2156	(%)	
Average age (SD)	25.4	(3.6)	26.8	(3.7)	26.1	(3.7)	< 0.001
**Age distribution**	**1083**		**1073**		**2156**		
18–19	41	(3.8)	15	(1.4)	56	(2.6)	< 0.001
20–24	445	(41.1)	293	(27.3)	738	(34.2)	
25–29	439	(40.5)	485	(45.2)	924	(42.9)	
30–34	158	(14.6)	280	(26.1)	438	(20.3)	
**University**	**1083**		**1073**		**2156**		
Completed Primary School	14	(1.3)	7	(0.7)	21	(0.9)	0.013
Completed Secondary School	111	(10.2)	73	(6.7)	184	(8.5)	
Incomplete Higher Education	172	(15.9)	179	(16.5)	351	(16.3)	
Completed Higher Education	786	(72.6)	814	(75.2)	1600	(74.2)	
**Marital status**	**1083**		**1073**		**2156**		
Married	221	(20.4)	349	(32.5)	570	(26.4)	< 0.001
Separated	14	(1.3)	13	(1.2)	27	(1.3)	
Single	823	(76.0)	685	(63.8)	1508	(69.9)	
No response	25	(2.3)	26	(2.4)	51	(2.4)	
**Employment status**	**1083**		**1073**		**2156**		
No employment	606	(56.0)	465	(43.3)	1071	(49.7)	< 0.001
Self employed	285	(26.3)	327	(30.5)	612	(28.4)	
Formal employment	160	(14.8)	257	(24.0)	417	(19.3)	
No response	32	(3.0)	24	(2.2)	56	(2.6)	

### Knowledge of COVID-19 symptoms and preventive measures

Knowledge on symptoms of COVID-19 was generally high, with most respondents being able to correctly identify an average of five symptoms of COVID-19, out of the ten examined. Female respondents were more likely to identify more symptoms correctly compared to men (*p* < 0.001). High fever (95.9%), difficulty in breathing (90.8%) and dry cough (83.5%) were the symptoms commonly mentioned, with the least being loss of taste (17.5%), loss of smell (15.3%) and diarrhea (12.1%).

A multivariate linear regression analysis was conducted to examine whether gender, education level and employment status predicts the level of awareness of signs and symptoms measured by total scores achieved. Female respondents had on average higher levels of awareness than males in identifying signs and symptoms compared to the male respondents [Coeff: 0.36; (*p* < 0.001), CI (0.19, 0.53)]. Those with college education had higher level of awareness of identifying signs and symptoms compared to those with lower levels of education [Coeff: 0.29; (*p* = 0.03), CI (0.10, 0.49)]. Consequently, young people in formal employment had higher levels of awareness of signs and symptoms compared to the unemployed [Coeff: 0.35; (*p* = 0.001), CI (0.15, 0.55)]. Notably, those who were in self-employment had on average lower levels of awareness of identifying signs and symptoms of COVID-19 compared to those in formal employment [Coeff: -0.15; (*P* = 0.191), CI (− 0.37, 0.07)].

In terms of knowledge of preventive measures, the most common preventive measures mentioned by the respondents were: washing hands with soap and running water (98.1%), use of hand sanitizers (95.6%), use of masks (93.1%), maintaining social distance of 1–2 m (88.0%) and staying home unless for urgent reasons (87.5%). On average, the youth mentioned at least 11 preventive measures correctly out of the 14 examined, with no significant differences by gender. When we examined the relationship between ability to identify preventive measures using multivariate linear regression analysis, there were no associations between gender, education, and employment status. However, young people in formal employment had on average higher levels of awareness of preventive measures compared to those without employment: [Coeff: 0.38, (*P* = 0.037) CI (0.02 0.74)]. Table 2 shows an analysis of knowledge levels of COVID-19 symptoms and preventive measures by the study respondents, and association with respondents’ characteristics.

**Table 2:** Knowledge of COVID-19 symptoms and preventive measures

% reporting the following symptoms	Female ***n*** = 1083	(%)	Male ***n*** = 1073	(%)	Total ***n*** = 2156	(%)	***P*** value
High Fever above 38	1053	(97.2)	1014	(94.5)	2067	(95.9)	0.001
Difficulty breathing	988	(91.2)	969	(90.3)	1957	(90.8)	0.13
Dry cough	**915**	(84.5)	**885**	(82.5)	1800	(83.5)	0.209
Headache	703	(64.9)	678	(63.2)	1381	(64.1)	0.404
Sore Throat	652	(60.2)	578	(53.9)	1230	(57.1)	0.003
Tiredness/fatigue	**558**	(51.5)	**480**	(44.7)	1038	(48.1)	0.002
Body Ache	343	(31.7)	**324**	(30.2)	667	(30.9)	0.459
Loss of taste	218	(20.1)	**160**	(14.9)	378	(17.5)	0.001
Loss of smell	186	(17.2)	**143**	(13.3)	329	(15.3)	0.013
Diarrhea	143	(13.2)	118	(11.0)	261	(12.1)	0.116
**Average no of signs (0–10) (SD)**	**5.3**	**(1.9)**	**4.9**	**(1.9)**	**5.1**	**(1.9)**	**< 0.001**
**% reporting the following ways of preventing COVID-19**	**1083**	**(%)**	**1073**	**(%)**	**2156**	**(%)**	***P*** **value**
Wash hands with soap and running water	1069	(98.7)	1046	(97.5)	2115	(98.1)	0.038
Use hand sanitizer	1042	(96.2)	1020	(95.1)	2062	(95.6)	0.19
Wear masks	1018	(94.0)	989	(92.2)	2007	(93.1)	0.095
Stand 1–2 m away from people	946	(87.3)	952	(88.7)	1898	(88.0)	0.326
Stay home unless urgent	961	(88.7)	925	(86.2)	1886	(87.5)	0.076
Do not touch face	956	(88.3)	921	(85.8)	1877	(87.1)	0.092
Do not shake hands	955	(88.2)	935	(87.1)	1890	(87.7)	0.462
Use digital money	867	(80.1)	838	(78.1)	1705	(79.1)	0.264
Do not go to weddings/funerals	771	(71.2)	739	(68.9)	1510	(70.0)	0.24
Do not go to church/mosque	763	(70.5)	730	(68.0)	1493	(69.2)	0.224
Reduce the number of people they come to contact with	723	(66.8)	713	(66.4)	1436	(66.6)	0.879
Avoid public transport/travelling	690	(63.7)	645	(60.1)	1335	(61.9)	0.085
Scrub/clean surfaces	711	(65.7)	584	(54.4)	1295	(60.1)	< 0.001
Get tested for coronavirus (COVID-19)	459	(42.4)	527	(49.1)	986	(45.7)	0.002
**Average scores (0–14) (SD)**	**11.0**	**(3.3)**	**10.7**	**(3.5)**	**10.9**	**(3.4)**	**0.104**
**Linear regression model**	**No of observations = 2156**			**P > F = 0.0000**		**R Squared = 0.0241**	
**Number of signs & symptoms**	**Coefficient**		**P value**		**Confidence interval**		
Gender (ref: Female)	0.36		< 0.001		0.19, 0.53		
Education; (ref: College education	0.296		0.008		0.070, 0.46		
Employment status: ref.: no employment							
Formal employment	0.35		0.001		0.15, 0.55		
Self-employed	-0.15		0.191		−0.37, 0.07		
**Linear regression model**	**No of observations = 2156**		**P > F = 0.0000**		**R Squared = 0.0241**		
**No of preventive measures identified**	**#of observations = 2156**		**P > F = 0.0536**		**R Squared = 0.0048**		
Gender (ref: Female)	−0.27		0.069		−0.021, 0.57		
Education; (ref: College education	0.10		0.543		−0.24, 0.46		
Employment status: ref.: no employment							
Formal employment	0.38		0.037		0.02 0 .74		
Self-employed	0.022		0.912		−0.37, 0.41		

### Perception of risk of COVID-19 infection

Among the young people, the perception of people at risk of infection with COVID-19 was varied, as illustrated on Table 3. Nearly 63.7 and 59.7% indicated that the elderly and those with weak immunity were at risk of infection. However, only 7.1% of young people reported that everyone was at risk of infection. When asked what the chances were of getting infected with COVID-19, about 29.0% perceived themselves to be at low risk, 38.9% at medium risk, and 2.7% reported no risk at all. Overall, 31.6% reported low or no risk at all with no differences between gender. Those who reported low or no risk gave various reasons for their responses, including: that they had not travelled (43.3%) or that God protects them (24.4%).

**Table 3:** Perception of risk of COVID-19 infections

	Female n = 1083	(%)	Male n = 1073	(%)	Total ***n*** = 2156	(%)	***P*** value
**% of respondents who reported the following as being at risk of COVID-19 infection**							
Elderly/over 50/over 60	681	(62.9)	692	(64.5)	1373	(63.7)	0.437
Sick/weak immune systems	657	(60.7)	631	(58.8)	1288	(59.7)	0.379
People with TB	438	(40.4)	444	(41.4)	882	(40.9)	0.658
People with HIV	424	(39.2)	438	(40.8)	862	(40.0)	0.429
Pregnant women	383	(35.4)	302	(28.1)	685	(31.8)	< 0.001
Children	278	(25.7)	252	(23.5)	530	(24.6)	0.239
Men	125	(11.5)	127	(11.8)	252	(11.7)	0.832
People in cold countries	114	(10.5)	131	(12.2)	245	(11.4)	0.218
Adolescents and youth	109	(10.1)	122	(11.4)	231	(10.7)	0.327
Women	93	(8.6)	88	(8.2)	181	(8.4)	0.747
Everyone	77	(7.1)	76	(7.1)	153	(7.1)	0.981
Dot know/ no response	6	(0.6)	10	(0.9)	16	(0.7)	0.307
**% of respondents who reported different risk levels (self) of COVID-19 infection**	**1062**		**1048**		2110		
Low risk	322	(30.3)	289	(27.6)	611	(29.0)	0.467
Medium risk	406	(38.2)	414	(39.5)	820	(38.9)	
High risk	267	(25.1)	288	(27.5)	555	(26.3)	
No Risk	29	(2.7)	27	(2.6)	56	(2.7)	
Do not know, no response	38	(3.6)	30	(2.9)	68	(3.2)	
Low or no risk	351	(33.1)	316	(30.2)	667	(31.6)	0.137
**Reason for perceived self-low risk**	**351**	**(%)**	**316**	**(%)**	**667**	(%)	
I am young	25	(7.1)	38	(12.0)	63	(9.4)	0.031
God protects me	74	(21.1)	89	(28.2)	163	(24.4)	0.034
The hot weather/climate	16	(4.6)	28	(8.9)	44	(6.6)	0.025
COVID is not in Africa/Kenya	1	(0.3)	1	(0.3)	2	(0.3)	0.941
I have not travelled	154	(43.9)	135	(42.7)	289	(43.3)	0.764
I am not a Caucasian	1	(0.3)	4	(1.3)	5	(0.7)	0.143
COVID is a lie	0	(0.0)	1	(0.3)	1	(0.1)	0.292
Do not know, no response	35	(10.0)	28	(8.9)	63	(9.4)	0.624
**Logistic regression model**	# of observations *n* = 2100						
**Perception of low or no Risk**	**OR**	***P*** **value**	**Confidence interval**				
Gender (ref: Female)	1.1	0.416	0.896		1.303		
Education; (ref: College education	0.85	0.174	0.689		1.06		
Employment status: ref.: no employment							
Formal employment	0.77	0.028	0.615		0.973		
Self-employed	0.75	0.033	0.588		0.978		

We computed multiple logistic regression analysis by examining the odds of those reporting being at any risk compared to those who did not perceive themselves to be at risk. There were no associations between those who reported any risk with gender [OR: 1.1, (*P* = 0.416) 95% CI (0.89, 1.30)] and education [OR: 0.85, (*P* = 0.174) 95% CI (0.68, 1.1)]. However, those in formal employment and those self-employed were less likely to report any risk compared to the unemployed [OR: 0.77, (*P* = 0.028), 95% CI (0.62, 0.97)] and [OR 0.76, (*P* = 0.033), 95% CI (0.58, 0.97)] respectively. Table 3 shows young people’s perception of risk of COVID-19 infection and an analysis of association with their characteristics.

**Fig. 1:**
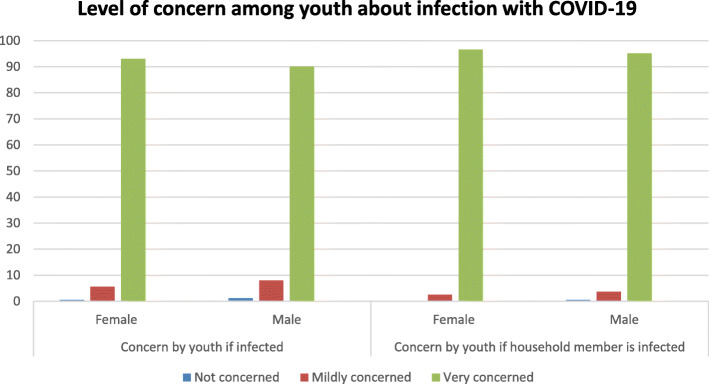
Level of concern among youth on infection with COVID-19

About 91.6% of the respondents (*n* = 1948) reported that they would be very concerned if they became infected with COVID-19. Similarly, almost all respondents (95.9%) reported higher levels of concern (*n* = 2042) if any of their household members was infected with the virus. Figure 1 shows the level of concern among youth on infection with COVID-19.

### Sources of information about COVID-19

Young people were asked how often they used various sources of information to stay informed about COVID-19. The response was on a scale of 1–4 with 1 being none, 2 - rarely, 3 - sometimes and 4 - all the times. Table 4 shows the proportion of young people who mentioned using each of the channels as sources of information for COVID-19. The most common sources which were used were social media sources (66.4%), followed by television programs (62.2%) and friends and internet at 49.8 and 49.3% respectively. The least used channels were community health workers (8.2%), church (5.8%), pharmacy and community meetings at 5.0 and 3.4% respectively.

**Table 4:** Use of various channels as sources of COVID-19 information

% reporting always using the following sources of information about COVID-19	Female ***n*** = 1074	(%)	Male ***n*** = 1066	(%)	Total ***n*** = 2140	(%)	***P*** values
Social media	728	(67.8)	692	(64.9)	1420	(66.4)	0.164
Television programs/shows	719	(66.9)	613	(57.5)	1332	(62.2)	< 0.001
Friends	567	(52.8)	499	(46.8)	1066	(49.8)	0.011
Internet	537	(50.0)	517	(48.5)	1054	(49.3)	0.018
Radio programs/shows	444	(41.3)	393	(36.9)	837	(39.1)	0.006
Spouse	270	(25.1)	365	(34.2)	635	(29.7)	< 0.001
Government SMS’s	284	(26.4)	282	(26.5)	566	(26.4)	0.884
Work colleagues	182	(16.9)	234	(22.0)	416	(19.4)	< 0.001
Acquaintances / neighbors	134	(12.5)	156	(14.6)	290	(13.6)	< 0.001
Posters/print advert	163	(15.2)	124	(11.6)	287	(13.4)	< 0.001
Books/magazines	151	(14.1)	111	(10.4)	262	(12.2)	0.008
Public health facility	116	(10.8)	135	(12.7)	251	(11.7)	< 0.001
NGO provider	126	(11.7)	121	(11.4)	247	(11.5)	0.15
Public announcement with megaphone	116	(10.8)	90	(8.4)	206	(9.6)	< 0.001
Private health clinic	70	(6.5)	90	(8.4)	160	(7.5)	0.001
Community health worker	75	(7.0)	101	(9.5)	176	(8.2)	< 0.001
Church	67	(6.2)	57	(5.3)	124	(5.8)	0.016
Pharmacy	45	(4.2)	61	(5.7)	106	(5.0)	0.055
Community meetings/spaces	36	(3.4)	37	(3.5)	73	(3.4)	0.013

### Adoption of preventive behaviors

**Fig. 2:**
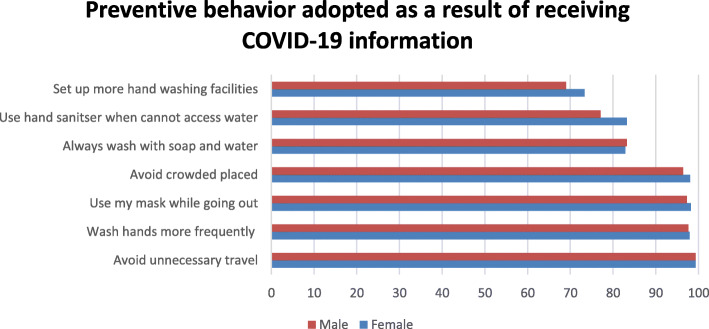
Preventive behaviors adopted as a result of receiving COVID-19 information

To assess adoption of preventive behaviors, young people were asked what they were doing differently since they started receiving messages about COVID-19. Figure 2 shows that most young people were adopting behavior necessary to slow down the infection rates. For example, nearly all respondents (99.3%) avoided unnecessary travel, 97.8% washed hands more frequently and 97.2% avoided crowded places. Among those that reported that they do not always wash hands with soap and water more frequently (*n* = 397), the main barriers mentioned were: they could not afford extra water (21.4%), or there was no water in the community (16.4%) or in the house (13.1%). Notably, 192 of those who reported not always washing hands with soap and water, reported no barrier at all, representing 48.4%.

The other important preventive measure was use of masks, with 97.7% reporting wearing masks while going out. When asked the type of mask they owned, 42.3% reported using surgical masks with more female reporting using such masks compared to men (*P* = 0.009) while majority (67.7%) use cloth masks. The key barriers to owning masks among those who mentioned that they do not own any were: ability to afford (51.1%), not knowing where to get one (35.6%) and discomfort (28.9%). Only 6.7% reported that they did not think masks work, while 2.2% indicated that they were not allowed to wear one. The third preventive measure that was assessed was the use of hand sanitizer if respondents could not access water and soap for hand-washing. This was reported among 80.2% of the youth. Among those who did not use a hand sanitizer (*n* = 420), 86.9% reported that they did not use it because it is very expensive, 19.8% said that hand sanitizers were not available in shops and less than 2.4% said they did not think hand sanitizers work. Figure 2 shows the preventive behaviors adopted by the youth as a result of receiving COVID-19 information.

### COVID-19 effects on access to health care services and socio-economic status

The study sought to find out the effects of COVID-19 on access to health care services and on the social and economic status of the youth, as illustrated on Table 5.

**Table 5:** Effects of COVID-19 on health care access, social and economic status

	Female ***n*** = 1083	(%)	Male ***n*** = 1073	(%)	Total ***n*** = 2156	(%)	***P*** values
**% of respondents who reporting not being able to access the following services**							
E-pills and other contraceptives	88	(8.1)	0	(0.0)	88	(4.1)	NA
Sanitary towels	117	(10.8)	0	(0.0)	117	(5.4)	NA
Condoms	26	(2.4)	156	(14.5)	182	(8.4)	< 0.001
Anti-retroviral treatment	19	(1.8)	30	(2.8)	49	(2.3)	0.105
HIV/AIDS counselling	33	(3.0)	69	(6.4)	102	(4.7)	< 0.001
Medicine for acute illnesses	92	(8.5)	124	(11.6)	216	(10.0)	0.018
Medicine for stomach/digestive problems	73	(6.7)	81	(7.5)	154	(7.1)	0.466
Medicine for diabetes/Blood pressure	30	(2.8)	39	(3.6)	69	(3.2)	0.254
Refills for other regular medications	99	(9.1)	93	(8.7)	192	(8.9)	0.699
Immunization/nutrition services for children	115	(10.6)	115	(10.7)	230	(10.7)	0.941
Medicine for pre-natal care	42	(3.9)	32	(3.0)	74	(3.4)	0.253
Medication of mental health/stress depression	69	(6.4)	102	(9.5)	171	(7.9)	0.007
**Other social effects:** **% of respondents who reported the below effects on social status**	**1083**	**(%)**	**1073**	**(%)**	**2156**	**(%)**	***P*** **values**
See friends less	440	(40.6)	436	(40.6)	876	(40.6)	0.998
Seeing family less	405	(37.4)	368	(34.3)	773	(35.9)	0.133
Stayed at home	391	(36.1)	319	(29.7)	710	(32.9)	0.002
Live in fear	326	(30.1)	321	(29.9)	647	(30.0)	0.925
Avoid public transport	306	(28.3)	339	(31.6)	645	(29.9)	0.09
Increased housework	163	(15.1)	101	(9.4)	264	(12.2)	< 0.001
More stress	314	(29.0)	257	(24.0)	571	(26.5)	0.008
Increased crime in neighborhood	108	(10.0)	149	(13.9)	257	(11.9)	0.005
Unable to access health care	142	(13.1)	113	(10.5)	255	(11.8)	0.064
Experienced more violence outside house	47	(4.3)	61	(5.7)	108	(5.0)	0.152
Stigma	23	(2.1)	37	(3.4)	60	(2.8)	0.062
Experienced more violence inside house	10	(0.9)	27	(2.5)	37	(1.7)	0.004
**Economic effects** **% of respondents who reported the below effects on economic factors**	**1083**	**(%)**	**1073**	**(%)**	**2156**	**(%)**	***P*** **values**
Significant reduction in income	464	(42.8)	615	(57.3)	1079	(50.0)	< 0.001
Increased expense in house	397	(36.7)	342	(31.9)	739	(34.3)	0.019
Increase in food prices	374	(34.5)	350	(32.6)	724	(33.6)	0.357
Complete loss of job	244	(22.5)	250	(23.3)	494	(22.9)	0.671
Unable to purchase sanitary towel	44	(4.1)	12	(1.1)	56	(2.6)	< 0.001

Young people were asked what health care services or medicines they were not able to access due to the COVID-19 pandemic. There were generally very low reported levels of inability to access certain services linked to SRH. For example, only 4.1% of the female respondents reported being unable to access emergency contraception (E-pills) and other contraceptives, 5.4% were not able to access sanitary towels while 8.4% were not able to access condoms. Additionally, only 2.3% were not able to access antiretroviral drugs (ARVs) while 7.9% were not able to access medication to relieve anxiety or depression.

In terms of social effects, 40.6% reported seeing friends less or family less (35.9%). Nearly a third (30.0%) reported living in fear, while 26.5% reported feeling stressed. Only 2.8% reported experiencing some form of stigma. However, COVID-19 was reported to have affected the youth economically with half of them (50.0%) reporting significant reduction in income with more male reporting this reduction in income compared to female (*p* < 0.001). About a third of the respondents reported increased expenses in their house (34.3%) or increased food prices (33.6%). Table 5 shows the effects of COVID-19 on access to health care services, and on the social and economic status of the youth.

## Discussions

Young people are a critical driver of economic and social change. Regardless of the nomenclature used, young people comprise the largest population in the developing world and comprise 27% of the world’s population [24, 25]. Despite being considered a healthy group, young people are at higher risk of sexual and reproductive morbidity and mortality [26–29]. They are also more likely to experience difficulties in obtaining specific and timely sexual and reproductive health (SRH) services and sexuality education, limiting their ability to realize SRH benefits. In Kenya, young people below the age of 25 years constitute 66% of the total population [30] and exhibit poor reproductive health outcomes such as teenage pregnancies, unmet need for contraception and early sexual debut [31]. To facilitate mechanisms of engaging young people in the COVID-19 response measures, we conducted a survey to provide information on the level of knowledge on symptoms, commonly used channels of information about COVID-19, adoption of preventive practices, perception of risk of infection and the effects of COVID-19 on access to specific health care services and other social and economic effects of the pandemic.

The survey showed high levels of knowledge on COVID-19 symptoms and prevention practices among the youth. Close to 90% of youth can correctly identify at least three symptoms of COVID-19. Of the 14 infection prevention methods listed, female respondents were able to correctly identify 71% of them and male respondents identified 73%, with no significant differences in the preventive methods mentioned. High levels of knowledge on symptoms and prevention strategies could be linked to two main reasons. First, the survey was conducted 4 weeks into the pandemic where the youth had been exposed to various messages on the Y-ACT online platform and other online platforms. The second reason could be linked to use of social media platforms with 66.4% reporting always using social media to access COVID-19 related information. Findings from a similar study showed that 45% of respondents had received information on COVID-19 using social media but were not necessarily trusted sources [23]. Use of influencers and experts on social media platforms could ensure provision of accurate information on COVID-19 and help shape the behavior of young people. This would have ripple effects as nearly half of our study respondents also mentioned using friends as sources of information. The high levels of knowledge on COVID-19 symptoms and prevention practices may lead to sustained adoption of preventive behaviors as was shown during the SARS outbreaks where high knowledge levels were linked to better adoption of precautionary practices, while clear communication and provision of updated information helped improve vigilance and preparedness during the pandemic [32].

Another important area of focus for the response team is messaging around perceived risks of infection. Our survey shows that 31.6% of the youth (1 in every 3) perceive themselves as being at low or no risk of getting infected with COVID-19. These levels varied with a previous study conducted in the informal settlement of Nairobi where about a third of participants felt they were at high risk of infection [23]. The low risk was associated with having no history of travel and the belief that God will protect them. Although history of travel is still a potential risk factor, breaking the community transmission would require strengthening messaging to address myths and misconceptions on risk of infection.

In terms of practice, majority of the youth begun adopting positive behavior practices to avoid infection since they started receiving messages on COVID-19. For example, the youth were avoiding unnecessary travel (99.3%), washing hands more frequently (97.8%) and using masks (97.7%). The few who were not practicing such behaviors reported lack of water or soap, the cost of masks, discomfort while wearing mask and costs of sanitizers as deterrents to practicing preventive behaviors. A previous survey conducted 2 weeks prior to this study, confirms that households are already performing risk reduction behaviors including increased hand washing with soap where possible, use of hand sanitizer, and staying home more [23]. Although young people reported practicing preventive behaviors, additional efforts should focus on how to sustain these behaviors on hygiene and social distancing. The response team should focus on ensuring that as the pandemic evolves, measures to strengthen quality assurance of preventive equipment such as cloth masks (that are being used widely) are put in place. The response team and the government should also provide guidance on proper use and re-use of cloth masks, including cloth specifications to ensure effectiveness of the masks in infection prevention.

Finally, our study has illustrated the effect of COVID-19 on access to specific health care services, social and economic effects. Four main emerging considerations are important. First, there were low proportions of respondents reporting inability to access contraceptives during the pandemic period (4.1%) and condoms at (8.4%). Inability to access Anti-retroviral treatment and HIV/AIDS counselling was relatively low at 2.3 and 4.7% respectively. This could be associated with the fact that the study was carried out within the first 2 months after the first COVID-19 case was detected in Kenya, hence health services might not have been heavily disrupted by the time of the study. Secondly, SRH services were listed by the Government as essential services during the pandemic period and continued to be provided in many health facilities. Despite low numbers reporting inability to access SRH services, there is need for innovative platforms to ensure access to health services, especially SRH for the youth, in the wake of the movement restrictions. Previous outbreaks indicate that when health systems are overwhelmed, mortality from vaccine-preventable and other treatable conditions are likely to increase drastically. For example, during the 2014–2015 Ebola outbreak, there was increased mortality caused by measles, malaria, HIV/AIDS, and tuberculosis attributable to health system failures. Deaths from these preventable conditions exceeded those from Ebola [20]. Other studies which examined the effect of Ebola on SRH showed a decline on use of family planning services [33].

The second consideration is that government guidelines and protocols on continuity of health services need to be disseminated widely especially among young people to assure them of their safety and available services. This is in line with the World Health Organization (WHO) operational planning guidelines that encourages countries to identify essential services, including routine vaccination; reproductive health services such as care during pregnancy and childbirth; management of mental health conditions and infectious diseases like HIV, malaria and TB, among others [34]. These services require prioritization by ensuring strategic shifts of limited resources to provide maximum benefit for the population [34]. Prioritization of services should be combined with a system that can track SRH service needs and use in different population segments to ensure targeted strategies are deployed not only to provide services but also to support dissemination and messaging around continuity of services. This is important especially among vulnerable populations such as young girls. Deploying effective measures early will help avoid long term consequences such as increased incidence of HIV infections and unwanted pregnancies.

The third consideration is associated with other social effects. The youth reported seeing friends and family less, but more notably, about a third reported living in fear, and a quarter feeling more stressed. This compounded with the fact that about 7.9% reported inability to access stress related medicines means that more investments in mental health programs and psychosocial support are needed during pandemics. Similar to the Ebola epidemic of 2014–2016, COVID-19 is expected to cause anxiety, depression and post-traumatic stress disorders, because of various factors, including physical distancing, stigma and discrimination, and job losses in many settings hardest hit by the pandemic [35]. Stakeholders need to explore the value of digital platforms as well as toll-free help lines in view of low digital literacy and low smartphone penetration in some areas, to ensure that people stay connected with their families and friends. Reporting and tracking of emerging mental health issues and responses being offered to the youth is also necessary. Investments that focus on effective social networks can be instrumental in supporting young people during the pandemic period.

The fourth consideration that requires strategic intervention is the loss of income reported by the youth, increased house expenses, cost of food and loss of jobs. Again, drawing from the 2014 Ebola outbreak, combining cash injections and skills training can stimulate employment and entrepreneurship. Government and partners therefore need to develop policies that will enhance resilience and recovery of small and medium sized enterprises, many of which are income channels for majority of the youth. Further, there is need for integration of skills development including alternative entrepreneurial skills within social protection programs. Additionally, expanding digital job opportunities for the youth can be a good avenue for alternative sources of income.

Several limitations and opportunities of this study are worth mentioning. First our study was among the first that examined the effect of COVID-19 among young people in Kenya. This provided us with an opportunity to assess the trajectory needed to advance interventions to support the national response. Although we do not have data disaggregated by urban or rural regions, we had some respondents completing the online survey while a substantial percentage were interviewed on phone after indicating inability to complete the survey online. In addition, our survey included all 47 counties in Kenya, indicating wider geographical coverage and inclusion of those who would have been disenfranchised by online access.

The second set of limitation is that our survey did not explore reasons for barriers to use of SRH health services during the pandemic and potential solutions. Further, while the study sought information on access to contraception and ARVs, we did not necessarily sample only sexually active youth, but rather all youth in general hence the responses to this questions might have been skewed to youth who did not necessarily need the services. Additional studies that reach a wider demography of youth in Kenya are needed, as well as studies that include the qualitative aspects of barriers and opportunities to improve access to SRH services during this period. However, by using rapid quantitative online survey, this study was able to provide guidance on appropriate information channels to reach young people as well as potential interventions needed to reach various youth population segments.

Lastly, while the team anticipated that all respondents would fill in the survey tool on their own, follow-up phone calls to respondents to take the survey revealed that a number of respondents were not able to complete the online tool on their own, mainly due to internet challenges. This necessitated phone-based interviews for 118 interviews out of the 2156 completed interviews. While our ESRC approval included written informed consent, the verbal consent provided by respondents was transferred to the survey tool (alongside their responses to the survey questions) as part of the phone interview process.

## Conclusion

Our study revealed high knowledge of COVID-19 symptoms, preventive strategies, and adoption of preventive practices. However, the socioeconomic effects of the pandemic suggest that risk communication should emphasize on continuity of health services and ways of implementing innovative interventions to meet the health and socioeconomic needs of the youth, to minimize the long-term consequences of the pandemic. Strategies to sustain behaviors positively adopted among the youth will be critical to reduce the spread of COVID-19. Lastly, we recommend tapping into the vast youth networks, for them to be ambassadors of behavior change and support dissemination of COVID-19 related information as they are a huge population segment spread across the country. Armed with proper personal protective equipment and information, the youth can support home-based care and ensure health facilities are not overwhelmed during this pandemic period.

## Supplementary Information

**Additional file 1.**

## Data Availability

All datasets analyzed during this study are included in the supplementary files (see supplementary file 2).

## Abbreviations

AIDS: Acquired Immunodeficiency Syndrome
ARV: Antiretroviral (drug)
COVID-19: Corona Virus Disease of 2019
ESRC: Ethics and Scientific Review Committee
HIV: Human Immunodeficiency Virus
MERS: Middle East Respiratory Syndrome
RAs: Research Assistants
SARS: Severe Acute Respiratory Syndrome
SARS-CoV-2: Severe Acute Respiratory Syndrome Coronavirus 2
SMS: Short Message Service
SRH: Sexual and Reproductive Health
STIs: Sexually Transmitted Infections
Y-ACT: Youth in Action
